# Green Tea Catechins Significantly Reduce Zika Virus in RBCs Through Viral Inactivation

**DOI:** 10.3390/pathogens15030334

**Published:** 2026-03-20

**Authors:** Xipeng Yan, Jinlian Li, Xiaoqiong Duan, Limin Chen, Yujia Li, Chunhui Yang

**Affiliations:** 1 Institute of Blood Transfusion, Chinese Academy of Medical Sciences and Peking Union Medical College, Chengdu 610052, China; xipeng.yan@pumc.edu.cn (X.Y.); xiaoqiongduan@163.com (X.D.); limin_chen_99@126.com (L.C.); 2 The Joint Laboratory on Transfusion-Transmitted Diseases (TTDs) between Institute of Blood Transfusion, Chinese Academy of Medical Sciences and Nanning Blood Center, Nanning Blood Center, Nanning 530007, China; jinlianli0902@163.com; 3 The Hospital of Xidian Group, Xi’an 710077, China

**Keywords:** Zika virus, green tea extract, viral inactivation, blood safety, red blood cell

## Abstract

Background: Despite significant improvements in blood safety, the risk of transfusion-transmitted infections persists, particularly from emerging and re-emerging viruses. For red blood cell (RBC) products, this risk is exacerbated by the fact that there is no routine testing for many of these pathogens, and effective, commercially available pathogen inactivation technologies specifically for RBCs are still lacking. This gap in the safety framework means that viruses capable of establishing an asymptomatic viremia—a characteristic of many arboviruses like Zika, dengue, and West Nile virus—present a tangible threat to the blood supply, highlighting the need for broad-spectrum countermeasures. Study Design and Methods: This study aims to investigate the antiviral activity of green tea extract (GTE) and its key catechins, epigallocatechin gallate (EGCG) and epicatechin gallate (ECG), against ZIKV in both cellular models and red blood cell (RBC) products. In vitro antiviral activity was assessed using A549 cells treated with GTE (150 μg/mL) or purified EGCG/ECG (20 μM). Mechanistic studies focused on viral attachment inhibition. Additionally, ZIKV-spiked RBC products were co-incubated with GTE (300 μg/mL) for 1 h to evaluate virucidal effects. Erythrocyte integrity was confirmed via hemolysis assays. Results: Co-treatment with GTE or catechins suppressed ZIKV replication by ≥3.64 logs (*p* < 0.001) in A549 cells. GTE and catechins primarily inhibited viral attachment. In RBCs, GTE reduced viral infectivity by 99.99% (4-log reduction) without compromising erythrocyte membrane integrity or cellular viability. Furthermore, RBCs with added GTE demonstrated a lower hemolysis rate during storage for up to 60 days. Conclusions: GTE exhibits potent virucidal activity against ZIKV in blood matrices, highlighting its potential as a pathogen reduction agent to enhance transfusion safety. Further development of GTE-based additive solutions or technologies is warranted.

## 1. Introduction

Currently, the residual risk of transfusion-transmitted (TT) viruses such as HIV, HBV, and HCV has been reduced to less than one per million following the widespread adoption of nucleic acid testing (NAT) [1]. For emerging TT virus-like ZIKV, a series of rapid response mechanisms—including NAT and Pathogen Reduction Technology (PRT)—must be established to address potential donations during the window period. After all, maintaining public trust in blood supply safety was considered paramount amid uncertainty.

Zika virus (ZIKV), a mosquito-borne flavivirus first identified in Uganda’s Zika Forest in 1947, has emerged as a global public health threat following its rapid spread across the Americas and the Asia–Pacific regions [2,3]. While transmitted primarily through *Aedes* mosquitoes and secondarily through sexual contact, vertical transmission, and blood transfusion, ZIKV poses significant challenges to blood safety systems. During the 2013–2014 epidemic in French Polynesia, 3% of samples from asymptomatic blood donors tested ZIKV-RNA positive [4]. Significantly higher prevalences of ZIKV (7.8%) were found in the epidemic period between 2020 and 2022 in Colombia [5]. Although approximately 80% of infections are asymptomatic, severe neurological sequelae such as congenital Zika syndrome (microcephaly incidence: 5–15% in prenatal exposure) and Guillain-Barré syndrome underscore the urgency of mitigating transfusion risks [6]. Notably, China’s current pre-transfusion screening protocols exclude ZIKV testing, creating potential vulnerabilities given the virus’ persistence in blood components for several months [7].

Conventional PRTs, such as methylene blue, amotosalen, and riboflavin-based photochemical treatments, provide broad-spectrum inactivation of viruses in plasma and platelets [8]. However, their inability to inactivate viruses in RBCs without increasing immunogenicity or compromising RBC function remains a critical limitation [9]. Furthermore, photochemical inactivation is hindered by the need for specialized equipment and the poor light penetration into packed RBC units. This gap necessitates exploration of novel virucidal agents compatible with RBC products.

Natural products are an important source of antiviral drugs. Among them, GTE has demonstrated significant antiviral capabilities in various studies. Especially against TT viruses. As the major antiviral component, epigallocatechin gallate (EGCG) was found to inhibit the replication step of HIV [10], DENV [11,12], and HBV [13,14] and the entry step of HCV [15], ZIKV [16], and CHIKV [14,17]. On the other hand, EGCG shows a protective effect on human erythrocytes by shielding them from oxidative damage [18,19,20]. However, no research has explored the antiviral effect of green tea components in blood products. Here, we evaluated GTE and its constituent polyphenols EGCG, ECG, and EGC for ZIKV inactivation efficacy in RBC suspensions, aiming to develop a phytochemical-based strategy for enhancing blood component safety.

## 2. Materials and Methods

### 2.1. Preparation and Compositional Analysis of Tea Extracts

In this study, GTEs were prepared by steeping 2 g of fresh green tea in 20 mL of double-distilled water at 98 °C for 10 min. After discarding the tea leaves, the solution was filtered through a 45 µm filter. The GTE was then diluted 20 times with double-distilled water for HPLC (Agilent Technologies, Santa Clara, CA, USA) analysis. The analysis was conducted using a C18 column (5 μm, 250 mm × 46 mm) at a flow rate of 1 mL/min and a column temperature of 35 °C.

In this study, GTE was prepared by steeping 2 g of fresh green tea in 20 mL of double-distilled water at 98 °C for 10 min. After discarding the tea leaves, the solution was filtered through a 0.45 μm filter. For HPLC analysis, 50 μL of the prepared GTE was mixed with 950 μL of stabilizer solution (detailed in Supplementary Table S1) to achieve a 20-fold dilution, and 10 μL of the diluted solution was subjected to analysis.

The HPLC analysis was performed using a C18 column (5 μm, 250 mm × 4.6 mm) at a flow rate of 1 mL/min and a column temperature of 35 °C. The UV detection wavelength was set at 278 nm with an injection volume of 10 μL. The gradient elution program was set as follows: 100% eluent A (detailed in Supplementary Table S1) for 10 min; 0–15 min, linear change from 100% A to 68% A and 32% B (detailed in Supplementary Table S1); 68% A and 32% B for 10 min, followed by re-equilibration with 100% A. After column temperature and flow rate were stabilized, 500 μL of sample solution was transferred into a sample vial, and 10 μL was injected for analysis.

### 2.2. Cells, Viruses, and Agents

A549 cells and Vero cells were maintained in our lab and cultured with Dulbecco’s Modified Eagle Medium (DMEM; Gibco) supplemented with 10% fetal bovine serum (FBS; HyClone, Logan, UT, USA) and 1% penicillin/streptomycin (Gibco, Grand Island, NY, USA) at 37 °C with 5% CO_2_. RBC units were obtained from healthy donors. All blood donors have given written consent allowing the donated blood to be used for testing and research. Our research was approved by the Institutional Review Board (Nanning blood center No. 2022-EC-05) and stored at 4 °C for ≤14 days. Zika virus (GZ01 strain, kept in our lab, passages 3–4) was propagated in A549 cells. Green tea extract (GTE) and purified catechins, including epigallocatechin gallate (EGCG), epicatechin gallate (ECG), and epigallocatechin (EGC) (purity > 99%; MedChemExpress, Monmouth Junction, NJ, USA), were dissolved in phosphate-buffered saline (PBS) to prepare stock solutions for experimental use. The GTE was derived from *Camellia sinensis* (family Theaceae, order Ericales), specifically from the Ganlu variety of green tea originating from Mount Mengding in Sichuan, China.

### 2.3. Cytotoxicity Assay

Cytotoxicity assay was measured using methods described by Ye et al. [21] with some modifications. The cytotoxicity of GTE in cells was assessed by CCK-8 assay according to the manufacturer’s instructions (Cell Counting Kit-8 C6005M; UElandy, Nanjing, Jiangsu, China). A549 cells were seeded in a 96-well plate at 1 × 10^4^ cells per well and cultured for 24 h and then treated with different concentrations of GTE for 24 h, with five replicates for each concentration. After 24 h of culture, 10 μL of CCK-8 solution per well was added, and the plate was further incubated for 1.5 h at 37 °C. The absorbance was measured at 450 nm. The 50% cytotoxic concentration (CC_50_) of GTE was calculated by GraphPad Prism 9.5 software (Graph-Pad Software, San Diego, CA, USA).

### 2.4. Quantitative Reverse Transcription-Polymerase Chain Reaction

Total RNA was extracted from ZIKV-infected A549 cells using TRIzol^®^ (Invitrogen, Carlsbad, CA, USA). cDNA synthesis was performed with 1 μg RNA using the PrimeScript™ RT Reagent Kit (Takara Bio Inc., Kusatsu, Shiga, Japan). ZIKV RNA levels were quantified via Roche Cobas z 480 real-time PCR system (Roche Diagnostics, Mannheim, Baden-Württemberg, Germany) with primers targeting the ZIKV NS5 gene [22]:

Forward: 5′-CAGCTGGCATCATGAAGAAYC-3′;

Reverse: 5′-CACTTGTCCCATCTTCTTCTCC-3′.

GAPDH was used as the endogenous reference gene for normalization, with the following primers:

Forward: 5′-GCCTCCTGCACCACCAACTG-3′;

Reverse: 5′-ACGCCTGCTTCACCACCTTC-3′.

Data were analyzed using the 2^−ΔΔCt^ method, with ZIKV replication expressed as fold change relative to untreated controls [23].

### 2.5. Plaque Forming Assay

The plaque-forming assay was based on a prior method described by Kim EA et al., with some modifications [24]. A prelaid monolayer of Vero cells was infected with ZIKV (MOI = 5, virus was pretreated with different concentrations of GTE/EGCG, etc.) for 2 h, followed by overlay with DMEM containing 1.3% methylcellulose. After 96 h, cells were fixed with 4% paraformaldehyde and stained with 0.1% crystal violet. Plaques were counted manually, and viral titers were calculated as PFU/mL.

### 2.6. Immunofluorescence Assay

ZIKV-infected A549 cells were fixed with 4% paraformaldehyde, permeabilized with 0.1% Triton X-100, and blocked with 5% BSA. Cells were incubated with anti-ZIKV capsid protein antibody (1:500; GeneTex, GTX133307, GeneTex Inc., Irvine, CA, USA) overnight at 4 °C, followed by Alexa Fluor 555-conjugated secondary antibody (1:1000; Invitrogen, Carlsbad, CA, USA). Nuclei were counterstained with DAPI for 15 min. Fluorescence images were captured using a Zeiss LSM 880(Carl Zeiss Microscopy GmbH, Jena, Thuringia, Germany) confocal microscope and analyzed with ImageJ (version 1.54g, National Institutes of Health, Bethesda, MD, USA).

### 2.7. Time-of-Addition Assay

A549 cells were treated with GTE following four sequential treatment protocols: pre-treatment (1 h prior to ZIKV exposure), co-treatment (concurrently during the 2 h viral adsorption phase), post-treatment (initiated 1 h post-infection and maintained for 24 h after viral internalization), and full-treatment (continuous exposure spanning from viral inoculation through experimental termination). Viral replication kinetics were subsequently evaluated through parallel methodologies combining RT-qPCR-based viral RNA quantification and plaque assay-based infectious virion enumeration.

### 2.8. Viral Attachment Assay

Under 4 °C conditions, a 12-well plate with confluent monolayer cells was pre-cooled for 1 h, then washed three times with pre-cooled PBS, and then virus and GTE were co-added to the cells. After incubation at 4 °C for 2 h, three times PBS wash was conducted. Then DMEM containing 1.3% methylcellulose was added, and the plate was incubated at 37 °C for 5 days until fixed with 4% paraformaldehyde and stained with 0.1% crystal violet.

### 2.9. Viral Internalization Assay

Similar to the attachment assay, a 12-well plate with confluent monolayer cells was pre-cooled at 4 °C for 1 h, then washed three times with pre-cooled PBS, and then the virus was added to the cells and incubated at 4 °C to let the virus bind. After incubation at 4 °C for 2 h, three times PBS wash was conducted to remove the unattached virus particles. After that, DMEM containing GTE was added and incubated at 37 °C for 1 h to let/inhibit virus internalization. After the last three times PBS wash, DMEM containing 1.3% methylcellulose was added, and the plate was incubated at 37 °C for 5 days until fixed with 4% paraformaldehyde and stained with 0.1% crystal violet.

### 2.10. Inactivation Assay

Virus inactivation assays were performed to determine whether the GTE directly affected virion stability. Stock solutions of ZIKV were incubated with GTE at various concentrations at 37 °C for 2 h. To avoid the effects of residual GTE in subsequent viral quantification, the drug-virus mixture underwent an 8-step serial ten-fold dilution, and viral titers were then determined using PFA. To evaluate the inactivation effect of GTE on ZIKV in blood components, we conducted experiments using washed red blood cells at 40% hematocrit. The experimental protocol involved spiking 0.9 mL of RBCs with 0.1 mL of ZIKV stock solution, followed by treatment with GTE. The drug-virus-RBC mixture underwent an 8-step serial ten-fold dilution and was inoculated onto pre-prepared Vero cell monolayers for plaque assay.

### 2.11. Hemolysis Rate Test

Fresh human whole blood (anticoagulated with citrate-phosphate-dextrose-adenine, CPDA-1) with a storage duration of <3 days was used. Erythrocytes were isolated by centrifugation at 1500× *g* for 5 min at 4 °C and washed three times with phosphate-buffered saline (PBS, pH 7.4) until the supernatant was colorless. After the final wash, the supernatant was discarded, and the packed erythrocytes were resuspended in CPDA-1 preservation solution for subsequent experiments. GTE, EGCG, ECG, or EGC was supplemented to the treatment group, while PBS was given to the control group. At days 0, 1, 10, 20, 30, 40, and 60 of storage, aliquots from each group were centrifuged at 4000× *g* for 10 min. The supernatant was carefully collected, and the supernatant hemoglobin concentration was measured at days 0, 1, 10, 20, 30, 40, and 60 using the Plasma Free Hemoglobin Assay Kit (Colorimetric Method) (Beijing Ruierda Biotechnology). Hemolysis rate (%) was derived using: %Hemolysis rate = [Free hemoglobin concentration × Volume of supernatant (mL)]/Total hemoglobin in erythrocytes.

### 2.12. Statistical Analysis

All quantitative data were expressed as mean ± standard deviation derived from three independent experiments. Statistical comparisons between groups were determined through one-way analysis of variance (ANOVA) followed by Tukey’s post hoc test for multiple comparisons. A probability threshold of *p* < 0.05 was considered statistically significant for all analyses. The complete statistical evaluation was conducted using GraphPad Prism software (version 9.5.1 for Windows, GraphPad Inc., San Diego, CA, USA).

## 3. Results

### 3.1. Preparation and Compositional Analysis of GTE

The tea extract was prepared through optimized aqueous extraction and subsequently diluted 20-fold for compositional analysis. Quantitative profiling of key bioactive compounds was performed via high-performance liquid chromatography (HPLC) following the national standard protocol (GB/T 8303-2018). The results revealed the following concentrations (mean values, *n* = 3) (Table 1).

**Figure 1:**
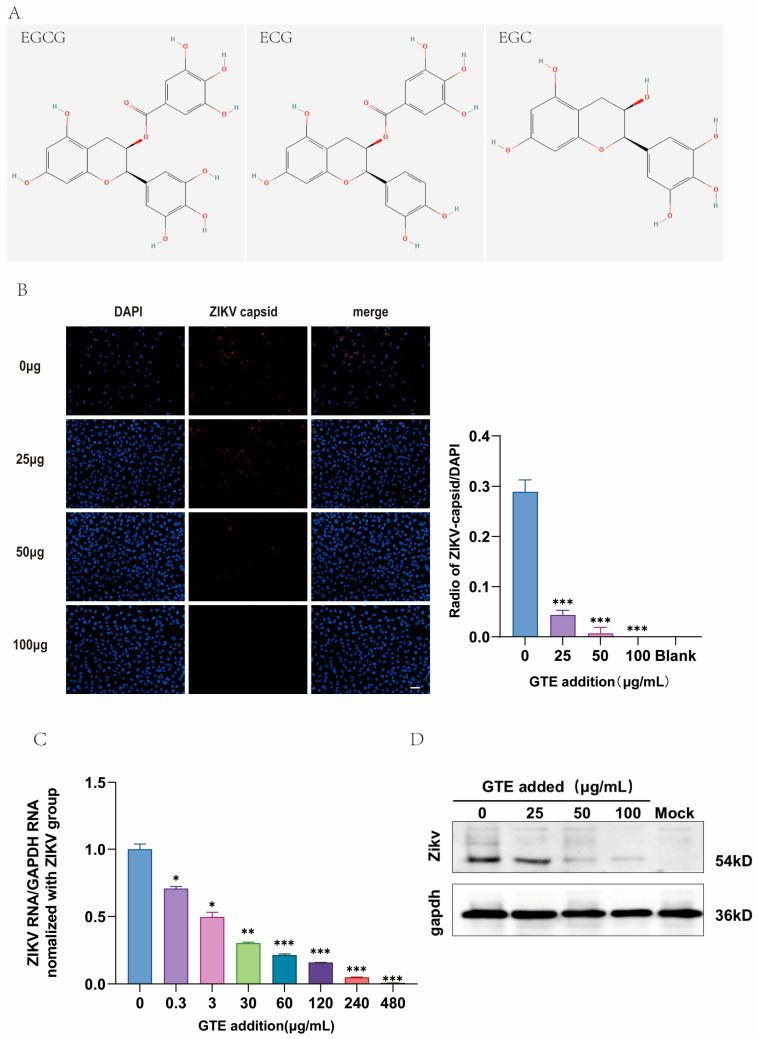
Inhibitory effect of GTE on ZIKV infection in A549 cells. (**A**) Structural formula of EGCG, ECG, and EGC. (**B**) Cells were immunostained with an anti-ZIKV capsid antibody (red) to visualize infected cells. Nuclei are shown in blue (DAPI). Original magnification, 200×; scale bar = 50 μm. The infection rate, defined as the ratio of ZIKV-positive (red) cells to the total number of DAPI-stained nuclei, was quantified from three replicate wells. (**C**) RT-qPCR analysis showing the relative expression of ZIKV RNA (normalized to GAPDH) in A549 cells treated with the indicated concentrations of GTE. (**D**) Western blot analysis shows that GTE treatment reduces the level of ZIKV capsid protein in infected cells. The graph presents the quantitative analysis of the protein bands normalized to the loading control β-actin. (* *p* < 0.05, ** *p* < 0.01, *** *p* < 0.001 vs. virus control).

Notably, EGC constituted the most abundant catechin derivative (19.9 mg/mL), followed by ECG (3.40 mg/mL) and EGCG (2.80 mg/mL). The absence of detectable free catechin (+C) aligns with typical green tea compositional profiles, where esterified catechins dominate. EGCG and ECG, two critical galloylated catechins, demonstrated moderate concentrations, collectively accounting for ~6.2 mg/mL in the extract. These compounds are recognized for their broad-spectrum antiviral activity, including potential inhibition of viral entry and replication (Figure 1A).

The high EGC content (19.9 mg/mL) suggests a preferential retention of non-galloylated catechins during extraction, which may synergize with galloylated derivatives to enhance bioactivity. Additionally, the presence of gallic acid (1.21 mg/mL) and caffeine (2.19 mg/mL) underscores the multifunctional nature of GTE, as these compounds contribute to antioxidant and anti-inflammatory effects [25]. This compositional profile provides a foundation for evaluating GTE’s efficacy against the Zika virus, particularly given the documented roles of EGCG and ECG in disrupting viral envelope integrity and modulating host–cell pathways.

### 3.2. GTE Presented Anti-ZIKV Effects

Quantitative reverse transcription polymerase chain reaction (qRT-PCR) analysis revealed that the IC_50_ value of the GTE against ZIKV was 3 µg/mL (Figure S2). A significant reduction in intracellular ZIKV RNA levels was seen from 60 μg/mL GTE and above, and treatment with 240 μg/mL GTE reduced ZIKV RNA levels by 95% (Figure 1C). Consistent with this, immunofluorescence assay (IFA) and Western blot (WB) results demonstrated a dose-dependent suppression of ZIKV capsid and envelope protein expression following GTE treatment (Figure 1B,D). As a premise of this assay, GTE concentrations used in these experiments showed no cytotoxic effects, as confirmed by the cell viability assay (Supplementary Figure S1). Collectively, these results demonstrate that GTE effectively inhibits ZIKV replication, as evidenced by the reduction in viral RNA and protein levels, progeny yield, and infection capability, without associated cytotoxicity.

### 3.3. GTE Inhibited ZIKV Growth in the Early Stage of Infection

**Figure 2:**
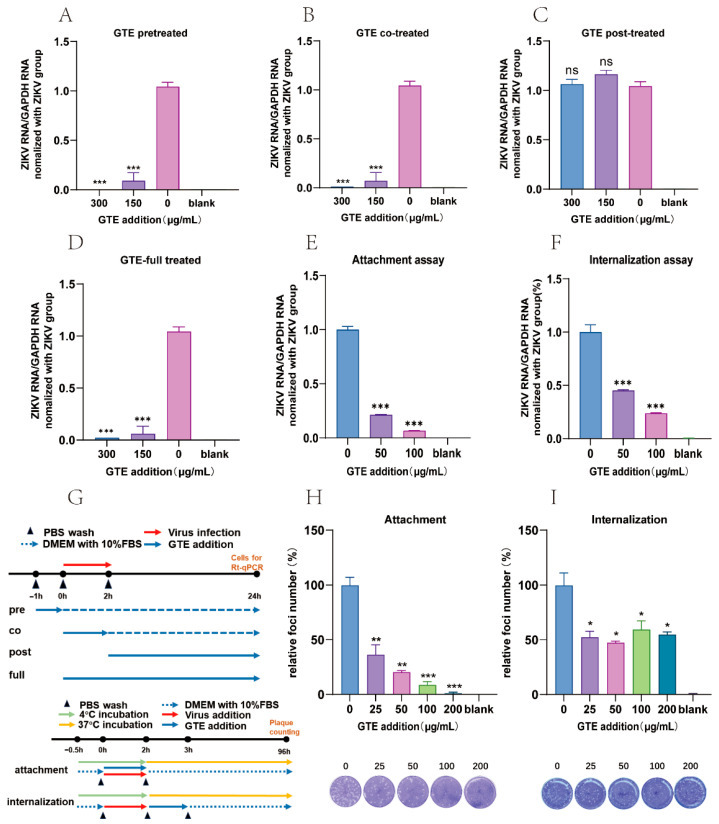
Time-of-addition assays define the antiviral stage of GTE. (**A**–**D**) RT-qPCR analysis of ZIKV RNA levels in A549 cells under different treatment timings: co-treatment, pre-treatment, post-treatment, and full-time treatment with GTE. (**E**,**H**) Attachment assay evaluating the effect of GTE on ZIKV binding to cells. (**F**,**I**) Internalization assay assessing the effect of GTE on ZIKV entry into cells. (**G**) Schematic diagram of the experimental procedures for the time-course assays. Data are expressed as the mean ± SD (*n* = 3). * *p* < 0.05, ** *p* < 0.01, *** *p* < 0.001, vs. control group. “ns” stands for no significant difference.

To investigate the temporal effects of GTE on ZIKV replication, a time course study was conducted. Four experimental groups were set up. In each group, cells were treated with 150 and 300 μg/mL GTE at different time points (300 μg/mL GTE shows no cytotoxicity): (i) pre-treatment (prior to viral infection), (ii) co-treatment (during 2 h viral adsorption), (iii) post-treatment (after viral infection), and (iv) full-treatment (throughout the process). Quantitative RT-PCR analysis at 24 h post-infection demonstrated dose-dependent inhibition, where GTE concentrations ≥ 150 μg/mL elicited ≥91% reduction in viral RNA load across pre-, co-, and full-treatment groups (*p* < 0.001). Notably, a 99% reduction was achieved at 300 μg/mL (Figure 2A–D). In contrast, post-treatment administration showed no statistically significant alteration in viral RNA levels (*p* > 0.05). These findings collectively indicate that GTE predominantly interferes with early-stage ZIKV replication events, rather than affecting post-entry viral processes.

### 3.4. GTE Suppressed ZIKV Infection by Inhibiting Viral Attachment

Virologists have found that a low temperature (4 °C) causes viruses to bind to cell surfaces without entering [26]. This is the principle used in attachment and internalization assays. GTE (100 µg/mL) treatment reduced ZIKV RNA by 93.7% and virus foci member by 91.3%, indicating that GTE treatment mainly interfered with ZIKV binding (Figure 2E,H). The entry assays revealed an inhibitory effect of 76.2% on ZIKV RNA and 50.1% on virus foci member (Figure 2F,I), further confirming that the antiviral effects of GTE are mainly associated with viral binding.

### 3.5. EGCG and ECG but Not EGC Inhibit the Replication of Zika Virus

**Figure 3:**
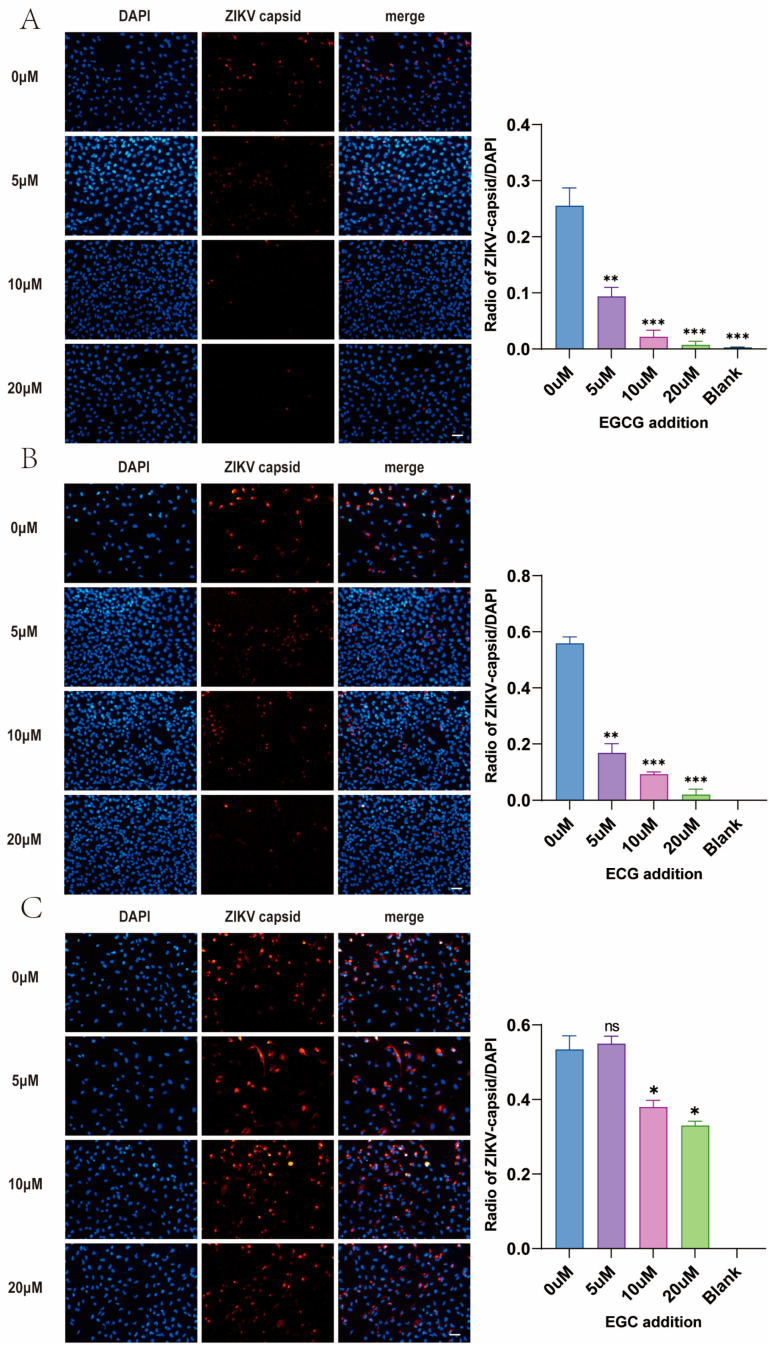
Inhibitory effects of EGCG, ECG and EGC on ZIKV infection in A549 cells. (**A**) Immunofluorescence analysis showing that EGCG significantly inhibits the expression of ZIKV capsid protein (red) in A549 cells; (**B**) Immunofluorescence analysis indicating that ECG remarkably suppresses the expression of ZIKV capsid protein (red) in A549 cells; (**C**) Immunofluorescence analysis presenting that EGC exerts a slight inhibitory effect on the expression of ZIKV capsid protein (red) in A549 cells. Nuclei were counterstained in blue with DAPI. Original magnification was 200×, and the scale bar represented 50 μm. The infection rate was quantified as the ratio of ZIKV-positive cells to the total number of DAPI-positive cells. Values are presented as the mean ± SD (n = 3). * *p* < 0.05, ** *p* < 0.01, *** *p* < 0.001 vs. the untreated infected control group; ns = not significant.

Based on this correlation and the remarked difference in antiviral potency, we inferred that EGCG and ECG might represent the key antiviral components in the green tea extracts. To test this hypothesis, we evaluated the individual compounds EGCG, ECG, and epigallocatechin (EGC) in viral inhibition assays (Figure 3A,B). The results demonstrated that EGCG and ECG (20 μM) exhibited potent inhibitory activity against ZIKV, thereby confirming their role as the primary antiviral constituents of the green tea extracts. In contrast, EGC exhibited a mild, dose-dependent inhibitory effect against ZIKV replication (Figure 3C).

### 3.6. GTE Inactivate ZIKV in RBC

ZIKV was reported to be transmitted through blood transfusion and can be actively tested in preserved RBC products [7]. To evaluate GTE’s inactivation effect on ZIKV in blood components, we performed experiments using washed RBCs at a 40% hematocrit—this value, lower than the 55–80% hematocrit of clinical RBC products, was selected to reduce suspension viscosity and ensure thorough washing of Vero cell monolayers, thus enabling accurate quantification of ZIKV titers. GTE concentrations (75, 150, and 300 μg/mL) were selected based on cytotoxicity: cell viability was >95% below 300 μg/mL (Figure S1), with 150 and 75 μg/mL as middle and low doses.

**Figure 4:**
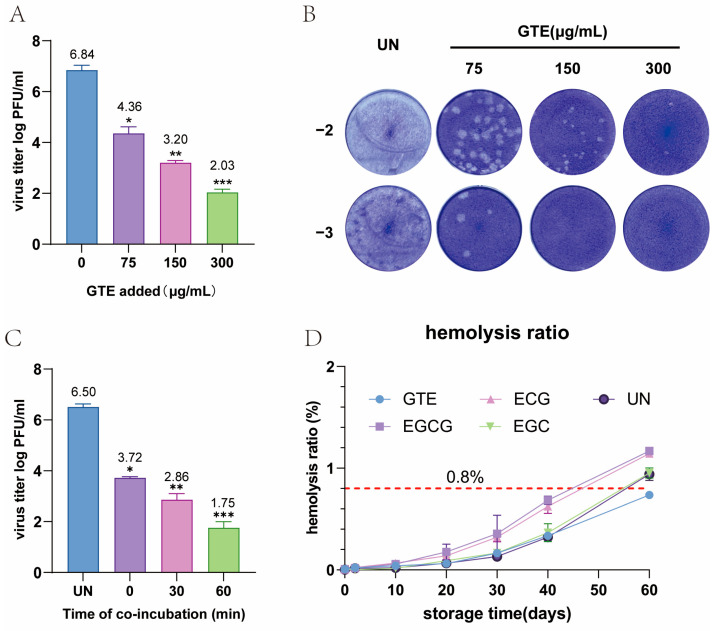
GTE inactivates ZIKV in RBCs without causing an increase in hemolysis rate. Plaque-forming assay demonstrating the concentration- and time-dependent virucidal effect of GTE in RBCs. The experimental protocol involved spiking 0.9 mL of RBCs with 0.1 mL of ZIKV stock solution, followed by treatment with GTE at concentrations of 75 μg/mL, 150 μg/mL, and 300 μg/mL. After 60 min of incubation at room temperature, the mixtures were inoculated onto pre-prepared Vero cell monolayers for plaque assay (**A**–**C**). The hemolysis rate test showed that GTE mildly decreases the hemolysis rate of RBCs during storage (**D**). The numerical values (log_10_ PFU/mL) are shown on the bars (**A**,**C**). * *p* < 0.05, ** *p* < 0.01, *** *p* < 0.001 compared to the untreated infected control.

Our results demonstrated a concentration-dependent antiviral effect of ZIKV. Treatment with 150 μg/mL GTE achieved a significant (≥3.64-logs) reduction in viral titer compared to untreated controls. Forceful viral inhibition (≥4-logs) was observed at the highest concentration tested (300 μg/mL) (Figure 4A–C). These findings confirm that GTE maintains its antiviral activity against ZIKV in RBC products.

### 3.7. GTE Decreases the Hemolysis Rate of Stored RBC Products

To evaluate the impact of GTE and its principal constituents on stored RBC quality [20], we conducted a longitudinal study over 60 days. Washed RBCs were treated with 300 µg/mL concentrations of GTE, epigallocatechin gallate (EGCG, 40 μM), epicatechin gallate (ECG, 40 μM), epigallocatechin (EGC, 40 μM), or control solution (CPDA-1) separately and then stored at 4 °C. Hemolysis rates were assessed at 10 days, 20 days, 30 days, 40 days, and 60 days. Throughout the initial 40 days of storage, hemolysis rates for all treatment groups remained below the 0.8% threshold mandated by the Chinese National Standard (GB18469). The GTE group demonstrated the most stable performance, consistently maintaining hemolysis rates below 0.8% (<0.8%). Furthermore, at the extended storage time point of day 60, only the GTE-treated RBCs continued to comply with the standard requirement, exhibiting a hemolysis rate of 0.742%. In contrast, the EGCG and ECG treatment groups showed significantly elevated hemolysis rates compared to the control group (*p* < 0.05), indicating a potential negative impact of these specific catechins on RBC membrane stability. While EGC treatment did not cause a statistically significant increase in hemolysis relative to the control, its protective effect was nonetheless inferior to that of GTE (Figure 4D).

## 4. Discussion

This study systematically evaluated the inhibitory effects of GTE and its primary active components against ZIKV, investigating their mechanisms of action and potential implications for blood safety.

The compositional analysis of GTE revealed that epigallocatechin gallate (EGCG, 10%), epicatechin gallate (ECG, 12%), and epigallocatechin (EGC, 70%) are the major constituents. However, only EGCG and ECG (below 40 μM) exhibited significant inhibition of ZIKV replication; the EGC showed negligible antiviral activity. This disparity likely stems from structural differences: EGCG and ECG possess galloyl groups that enable direct interactions with viral envelope proteins (e.g., ZIKV E protein) or host receptors via hydrogen bonding and hydrophobic forces, whereas EGC lacks this functional moiety. These results align with prior studies highlighting the critical role of galloylation in the antiviral potency of catechins against flaviviruses [27]. Furthermore, the synergistic inhibition of ZIKV NS2B-NS3 protease by EGCG and ECG may enhance their antiviral efficacy [12]. This underscores that GTE’s activity depends not only on total polyphenol content but also on the structural specificity of its components.

A time-course study demonstrated that GTE effectively suppresses ZIKV replication when administered during the pre-treatment, co-treatment, or full-course treatment phases, but not during post-infection. This indicates that GTE primarily targets early stages of the viral lifecycle, such as viral attachment and entry. Binding and entry assays further clarified this mechanism: GTE inhibited ZIKV binding to host cells in a dose-dependent manner, whereas its effect on viral entry was “non-dose-dependent”. These observations suggest two potential modes of action. 1. Competitive binding: EGCG and ECG may block ZIKV E protein from interacting with host receptors (e.g., Axl or TIM-1) by occupying key binding domains (e.g., Asn154, Thr156) [16]. 2. Non-specific entry interference: GTE might alter virus membrane fluidity or form a physical barrier to impede viral internalization. This multi-modal mechanism enables GTE to achieve significant antiviral effects at low concentrations (100 μg/mL), highlighting its practical advantage for therapeutic applications.

A new finding of this study is that GTE and its active components (EGCG/ECG) can inactivate ZIKV in RBC preparations, rendering the virus non-infectious. Our results showed that at concentrations below 300 μg/mL (maximal non-cytotoxic dose), ZIKV was reduced by more than −4 logs. This has critical implications: mitigating transfusion-transmission risks. ZIKV remains viable in RBCs for weeks, posing a threat to blood safety [28]. Conventional PRTs, such as methylene blue, amotosalen, and riboflavin-based photochemical treatments, often target the nucleic acid of pathogens and may compromise RBC functionality [29,30]. The S-303, combined with the glutathione treatment regimen, once showed promise, but during clinical trials, it was discovered that some subjects developed antibodies against the S-303-treated red blood cells. Therefore, this method was also limited [31]. In contrast, GTE inactivates ZIKV while preserving RBC membrane integrity (hemolysis rates remain stable), offering a natural and biocompatible alternative for blood additive solutions [32]. Our data suggest that the synergistic interaction of constituents within GTE is more effective in preserving RBC membrane integrity than any single catechin component tested.

Given the demonstrated efficacy of EGCG/ECG against other transfusion-transmissible viruses (e.g., HBV, HCV, DENV) [12,33], GTE may serve as a universal viral inactivation agent for RBC. However, the in vivo persistence and metabolic fate of GTE following transfusion remain to be fully elucidated. As a potential pharmaceutical, its long-term stability, bioavailability, and sustained activity in the circulation require further characterization. From a public health and sustainable pharmaceutical development perspective, establishing the safety, durability, and scalability of GTE-based viral inactivation is critical to support its future translation into clinical and industrial settings.

While this study provides compelling evidence, several challenges remain: We have preliminarily confirmed that GTE can inactivate ZIKV in RBC units and maintain better cell integrity during long-term storage. However, we have not yet determined whether the inactivation effect of GTE remains effective within the RBC cytomembrane. This limitation can be partially explained, and its impact weakened, by the existing evidence that ZIKV primarily infects nucleated blood cells (e.g., monocytes) via receptor-mediated endocytosis [34]. Since mature RBCs lack both the cellular machinery required for viral replication and ZIKV receptors, we hypothesize that ZIKV exists predominantly as a free virus or adsorbed to RBCs in stored RBC preparations. As demonstrated in Section 3.3 and Section 3.4, GTE effectively inactivated both free and adsorbed virus. To address this gap, future work will prolong incubation in virus titration to allow potential intracellular virus redistribution and use RBC freeze–thaw lysis to measure intracellular titers, accounting for freeze–thaw effects and hemoglobin cytotoxicity on Vero cells.

Furthermore, preclinical in vivo transfusion studies based on animal models remain an indispensable step toward the application and development of the GTE-based virus reduction strategy. To further validate the translational potential of GTE as a viral inactivation agent for blood products, subsequent comprehensive studies will be focused on two core aspects: first, to systematically evaluate the impacts of GTE on RBC functionality and safety, including detailed detection of critical physiological and functional parameters such as RBC oxygen-carrying capacity, energy metabolism, and membrane deformability during long-term storage; second, to expand the research scope of viral pathogens and verify the virucidal effectiveness of GTE against a variety of clinically important transfusion-transmissible viruses (e.g., HBV, HCV, DENV). These in-depth studies will provide more sufficient experimental evidence for the development of GTE as a safe and effective broad-spectrum viral inactivation strategy in transfusion medicine.

Regarding the clinical safety of GTE-mediated viral inactivation in RBCs, the final concentration of GTE in the transfusion product is 300 μg/mL, corresponding to approximately 150 mg per 2-unit transfusion. After systemic dilution, the estimated peak plasma concentration is approximately 30 μg/mL. Current safety data and acceptable daily intake limits for catechins are derived from oral administration and cannot be directly extrapolated to transfusion, which bypasses hepatic first-pass metabolism and results in higher bioavailability [35,36,37]. Therefore, the safety of this concentration requires further validation. To improve safety, the GTE concentration can be reduced, since our results demonstrated that 100 μg/mL GTE still significantly reduced ZIKV infectivity. In addition, GTE could be removed by filtration after viral inactivation to further enhance transfusion safety.

This study establishes that GTE inhibits ZIKV by targeting viral attachment, with EGCG and ECG identified as the key active components. Crucially, GTE inactivates ZIKV in RBCs without compromising cellular function, offering a novel strategy to enhance blood safety—particularly in resource-limited regions and during emerging arboviral outbreaks. Future work should prioritize in vivo validation, formulation optimization, and expansion to other transfusion-relevant pathogens, accelerating the translation of GTE from bench to bedside.

## Figures and Tables

**Table 1:** Composition of GTE.

Component	Abbreviation	Concentration (mg/mL)	Percentage of Total Polyphenols
- Epigallocatechin	EGC	19.90 ± 1.20	69.24 ± 4.18%
- Epicatechin	EC	1.43 ± 0.09	4.98 ± 0.31%
- Epigallocatechin gallate	EGCG	2.80 ± 0.06	9.74 ± 0.21%
- Epicatechin gallate	ECG	3.40 ± 0.11	11.83 ± 0.38%
- Gallic acid	GA	1.21 ± 0.08	4.21 ± 0.28%
- Total Polyphenols		28.74 ± 1.91	100%
- Caffeine	-	2.19 ± 0.13	-

## Data Availability

The raw data supporting the conclusions of this article will be made available by the authors on request due to privacy.

## Supplementary Materials

The following supporting information can be downloaded at: https://www.mdpi.com/article/10.3390/pathogens15030334/s1. Figure S1. Cytotoxicity of GTE on A549 cells. Cell viability was determined by the A549 cell viability assay after treatment with various concentrations of GTE (0–1360 μg/mL) for 24 h. The 50% cytotoxic concentration (CC_50_) was calculated as 908.8 μg/mL. Data are presented as mean ± standard deviation (SD). Figure S2. Inhibitory effect of GTE on ZIKV virus.The inhibition ratio was determined after treatment with various concentrations of GTE (0–500 μg/mL). The half-maximal inhibitory concentration (IC_50_) was calculated as 3 μg/mL. Data are presented as mean ± standard deviation (SD). Figure S3. HPLC chromatograms of standard compounds (GA, EGC, caffeine, EC, EGCG, ECG) and GTE (detection at 278 nm). Peaks correspond to GA (≈2.9 min), EGC (≈3.5 min), caffeine (≈11.5 min), EC (≈14.7 min), EGCG (≈13.7 min), and ECG (≈21.8 min), verifying the phe-nolic composition of GTE. Table S1. Preparation of HPLC Solutions and Catechin Standard Solutions.

## Abbreviations

RT-qPCR	Reverse Transcription-Quantitative Polymerase Chain Reaction
GTE	Green Tea Extract
EGCG	Epigallocatechin Gallate
ECG	Epicatechin Gallate
EGC	Epigallocatechin
RBCs	Red Blood Cells
PRTs	Pathogen Reduction Technology
HIV	Human Immunodeficiency Virus
HBV	Hepatitis B Virus
HCV	Hepatitis C Virus
DENV	Dengue Virus
WNV	West Nile Virus
ZIKV	Zika virus
CHIKV	Chikungunya Virus
HPLC	High-performance liquid chromatography
MOI	Multiplicity of Infection
CPDA	Citrate-Phosphate-Dextrose-Adenine
