# Supplementary material for: Green Tea Catechins Significantly Reduce Zika Virus in RBCs Through Viral Inactivation
Source: Pathogens. 2026 Mar 20;15(3):334. doi: 10.3390/pathogens15030334 (PMC13028612; doi:10.3390/pathogens15030334)
Supplement: Supplementary file 1 [file pathogens-15-00334-s001.zip › pathogens-4166310-Supplementary Materials.pdf]

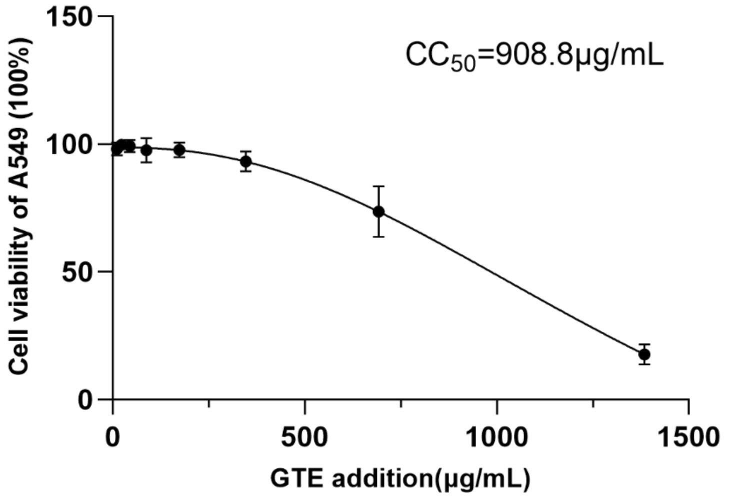

Figure S1. Cytotoxicity of GTE on A549 cells. Cell viability was determined by the A549 cell viability assay after treatment with various concentrations of GTE (0–1360 μg/mL) for 24 h. The 50% cytotoxic concentration (CC_{50}) was calculated as 908.8 μg/mL. Data are presented as mean ± standard deviation (SD).

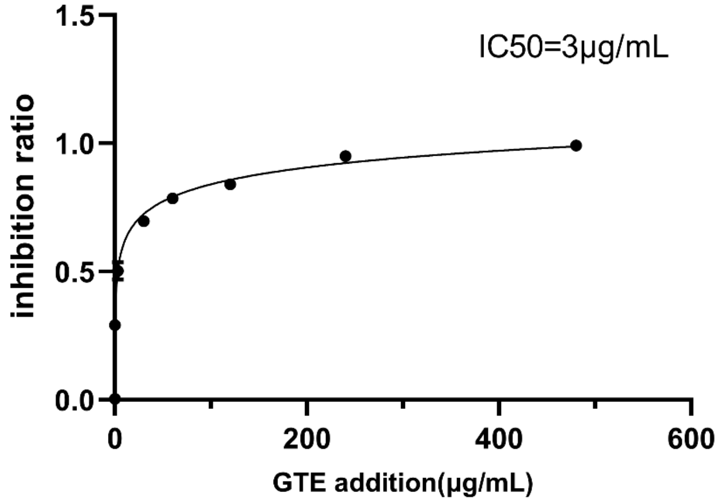

Figure S2. Inhibitory effect of GTE on ZIKV virus.The inhibition ratio was determined after treatment with various concentrations of GTE (0–500 μg/mL). The half-maximal inhibitory concentration (IC_{50}) was calculated as 3 μg/mL. Data are presented as mean ± standard deviation (SD).

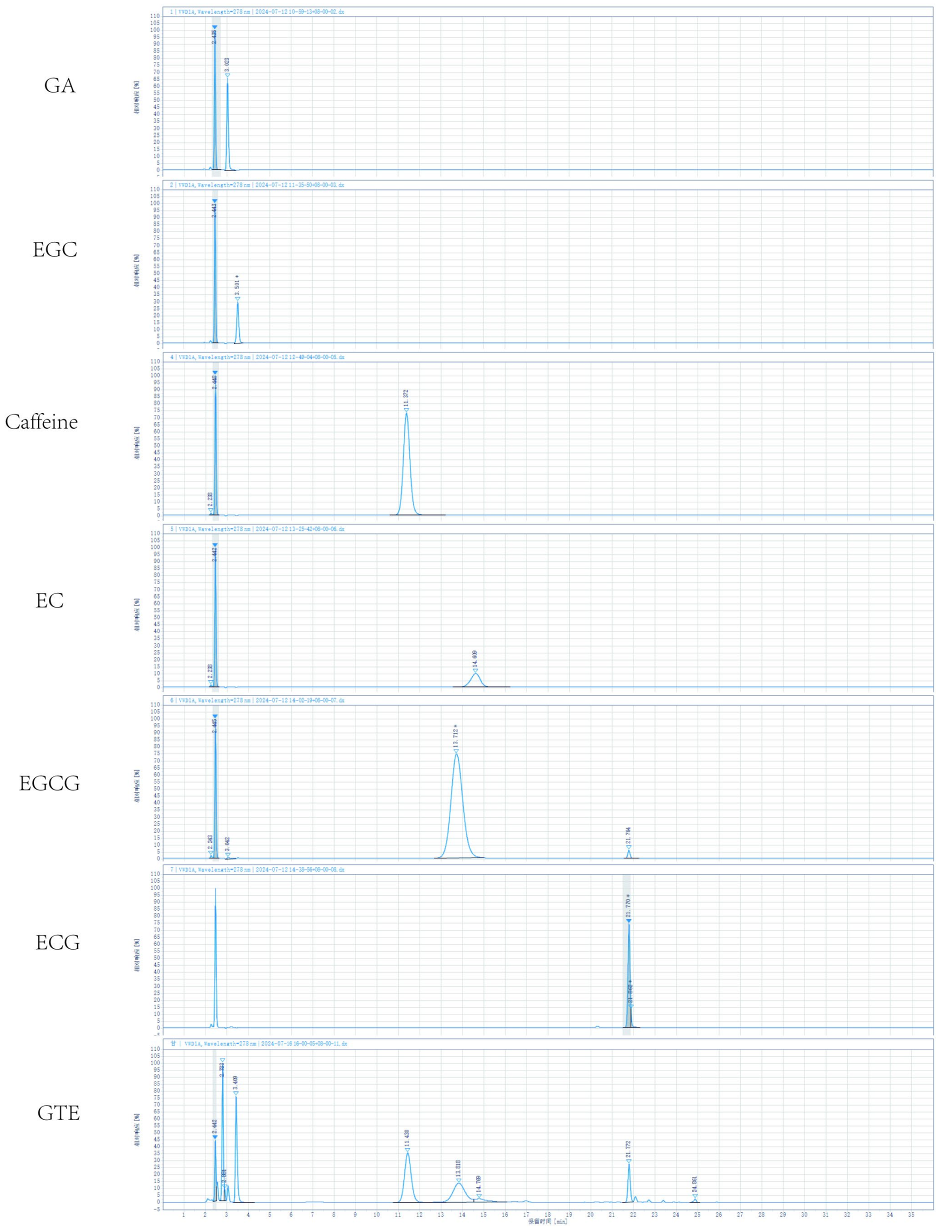

Figure S3. HPLC chromatograms of standard compounds (GA, EGC, caffeine, EC, EGCG, ECG) and GTE (detection at 278 nm). Peaks correspond to GA (≈2.9 min), EGC (≈3.5 min), caffeine (≈11.5 min), EC (≈14.7 min), EGCG (≈13.7 min), and ECG (≈21.8 min), verifying the phe-nolic composition of GTE.

Supplementary Table S1: Preparation of HPLC Solutions and Catechin Standard Solutions

Solution Type	Composition and Preparation
Reagents Prepared in Advance	Chromatographically pure acetonitrile, methanol, acetic acid, 10 mg/mL EDTA solution, 10 mg/mL ascorbic acid solution
Mobile Phase A	90 mL acetonitrile + 20 mL acetic acid + 2 mL EDTA solution, made up to 1000 mL with

	ultrapure water, filtered through 0.45 μm membrane
Mobile Phase B	800 mL acetonitrile + 20 mL acetic acid + 2 mL EDTA solution, made up to 1000 mL with ultrapure water, filtered through 0.45 μm membrane
Stabilizer Solution	25 mL EDTA solution + 50 mL acetonitrile + 25 mL ascorbic acid solution, made up to 500 mL with ultrapure water
Catechin Standard Solutions (prepared with stabilizer solution)	Caffeine: 100 μg/mL; Gallic acid (GA): 20 μg/mL; Epicatechin (EC): 100 μg/mL; Epigallocatechin (EGC): 200 μg/mL; (+)-Catechin (+C): 100 μg/mL; Epigallocatechin gallate (EGCG): 250 μg/mL; Epicatechin gallate (ECG): 150 μg/mL
